# Beneficial effect of hydrolyzed egg in allergy

**DOI:** 10.1186/2045-7022-1-S1-P101

**Published:** 2011-08-12

**Authors:** Sophie Nutten, Antoine Wermeille, Sébastien Holvoet, Alexandre Panchaud, Fériel Hacini-Rachinel, Guénolée Prioult, Rodolphe Fritsché, Annick Mercenier

**Affiliations:** 1 Nestlé Research center, Nutrition and Heath, Lausanne, Switzerland; 2 Nestle Research Center, Lausanne, Switzerland

## 

There is clinical evidence to recommend the use of partially hydrolyzed infant formulas (HA-IF) for at risk children as an option for prevention of allergic diseases, particularly atopic dermatitis/eczema (Szajewska et al. 2010). As cow's milk allergic infants are at risk of developing allergy to newly introduced foods at weaning, we aimed to extend the concept of HA-IF to egg, another potentially allergenic food.

Hydrolyzed egg (HA egg) was produced using a specific combination of heat and enzymatic treatments of whole egg. Characterization and reproducibility of the product was assessed using Size Exclusion Chromatography. Residual antigenic proteins (ovalbumin and ovomucoid) were quantified using ELISA. Allergenicity of the HA egg was tested both in vitro (serotonin release assay) and in vivo (rat model of allergy to ovalbumin). The capability of HA egg to induce oral tolerance to ovalbumin was assessed in both rat and mouse models.

HA egg production was shown to be highly reproducible in terms of peptide profiling, residual allergenic proteins and in vivo benefits. Composition of HA egg, as compared to whole egg showed a clear shift towards peptides <1000 Da. The content of major allergenic proteins was reduced by at least 1000 fold in comparison to whole egg. The allergenicity of HA egg was also highly reduced in vitro and almost no allergic response (assessed by RMCPII quantification in sera) was observed in rats preliminary sensitized to ovalbumin and orally challenged with HA egg. Moreover, we showed that HA egg is able to induce oral tolerance to ovalbumin when used in similar quantity as whole egg.

In conclusion, we have shown that the concept of "low allergenicity linked to induction of oral tolerance" can be applied to other food allergens than cow's milk. A small human SOTI trial with HA eggs is being launched.

